# Peak Integration of Electropherograms in GMP and Research Labs: Navigating Increased Scrutiny Amid Data Integrity Audits and Inspections

**DOI:** 10.1002/elps.70002

**Published:** 2025-06-19

**Authors:** Timothy Blanc, Hermann Wätzig, Cari Sänger ‐ van de Griend

**Affiliations:** ^1^ Technical Services/Manufacturing Science Eli Lilly and Company Branchburg New Jersey USA; ^2^ Institute of Medicinal and Pharmaceutical Chemistry Technische Universität Braunschweig Braunschweig Germany; ^3^ Kantisto BV Baarn The Netherlands; ^4^ Department of Medicinal Chemistry Faculty of Pharmacy Uppsala Universitet Uppsala Sweden

## Abstract

Capillary electrophoresis (CE) often offers superior separation relative to chromatography for macromolecules like monoclonal antibodies (mAbs), a major pharmaceutical class. However, electropherogram baselines pose challenges that traditional chromatography algorithms cannot address, requiring complex integration processes. Integration in good manufacturing practice (GMP) laboratories is critically important and has become a focus of data integrity‐centric regulatory inspections. Electropherogram integration challenges, the increased use of CE, data systems developed for chromatograms rather than electropherograms, and the increased regulatory scrutiny call for a resolution. This necessity also extends to R&D, clinical, and academic labs. This review examines authoritative sources such as pharmacopoeias, World Health Organization (WHO), Parenteral Drug Association (PDA), and scientific literature. However, none address electropherogram integration. These sources concur that companies should develop integration policies and SOPs. Training programs must ensure analysts are proficient in integration techniques and reviewers are appropriately qualified to assess integrations. Integration parameters must be captured, including slope sensitivity, smoothing factors, and timed events like peak start and stop and baseline correction. Analytical procedures (APs) should include illustrations that define proper integration. Although automatic integration is preferred for its efficiency and objectivity, it is not always accurate. Therefore, manual integration should be permitted under specific conditions, with all settings and iterations documented, justified, and reviewed. Industry collaboration is proposed to create practical integration guidelines specifically for CE.

## 1 Introduction

Protein analysis has long relied upon sodium dodecyl sulfate polyacrylamide gel electrophoresis (SDS‐PAGE) as a cornerstone analytical technique. However, limitations in quantitative analysis due to an indirect detection system and cumbersome gel archival methods prompted the development of capillary electrophoresis (CE) methodologies, particularly SDS‐capillary gel electrophoresis. This technique, known as CE‐SDS, has evolved from SDS‐PAGE into a more automated and quantitative method. Given the rise of monoclonal antibodies (mAbs) as a predominant class of biotherapeutics, CE‐SDS gained popularity, becoming, perhaps, the most‐used CE method in both good manufacturing practice (GMP) environments and biopharmaceutical research settings.

**FIGURE 1:**
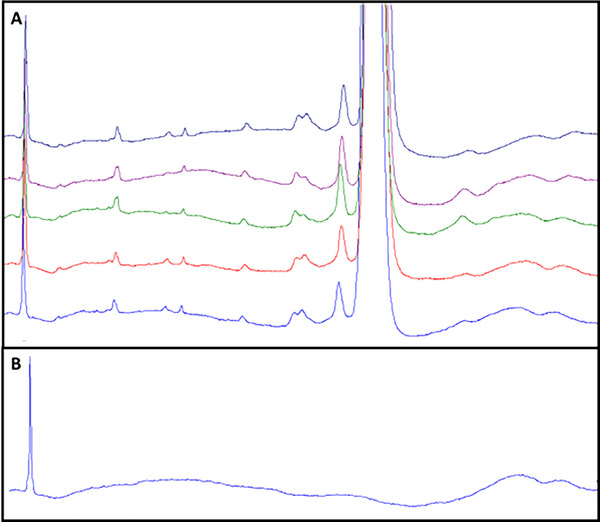
Non‐reduced CE‐SDS example of a common GMP application of capillary electrophoresis and challenges of automatic integration: impact of baseline drift and noise. (A) Five successive analyses of a therapeutic monoclonal antibody (mAb). (B) A blank injection to demonstrate the shape of the baseline due to drift and noise. Parts (A and B) illustrate the uneven baselines that are characteristic of CE‐SDS and emblematic of capillary electrophoresis in general. Additionally, in (A), it also illustrates the inconsistency of the baseline. The baseline shape and inconsistency are formidable challenges for automatic integration CDS Functions.

**FIGURE 2:**
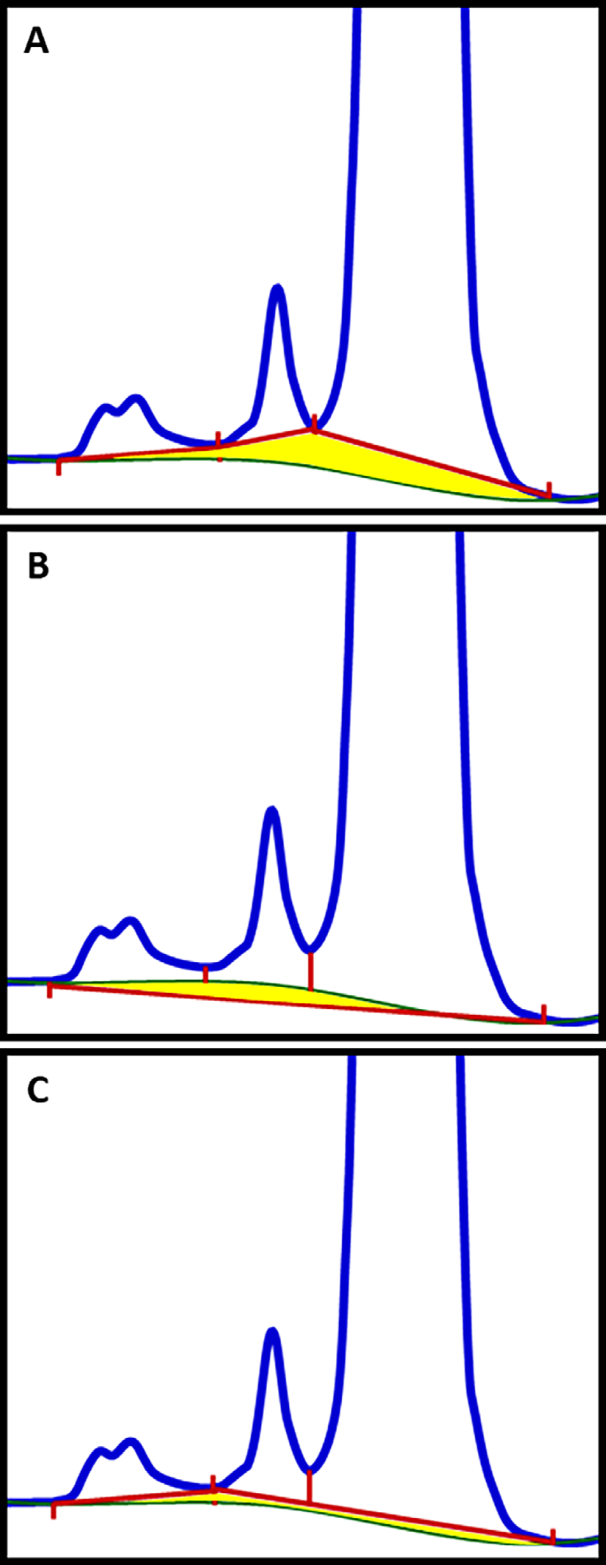
Non‐reduced CE‐SDS example of a common GMP application of capillary electrophoresis and challenges of automatic integration: impact of integration approaches on accuracy, detail of Figure 1. The region between the integration points and the baseline (shaded in yellow) are accentuated to aid in visualizing the area that might be either underreported (A and C) or overreported (Figure 1B). A lesser extent of yellow shading indicates a more appropriate integration. The integration markings (highlighted in red) in conjunction with the baseline reveal that (B), which employs a hybrid approach of “valley to valley” and “common baseline,” offers the most accurate integration. Although which integration is most correct or accurate may be debatable, laboratories should practice consistency in their integration approach not only through the sequence but from run to run. This figure underscores the significance of considering the baseline when determining the suitability of the final integration.

**FIGURE 3:**
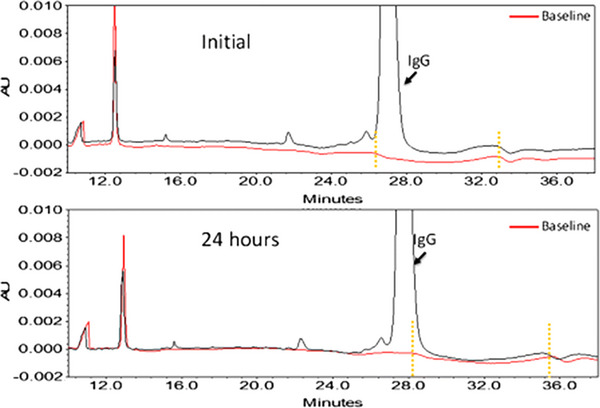
Experimental data showing systematic drift and quantification inconsistency in a CE‐SDS non‐reducing assay. Electropherograms of IgG drug overlaid with the corresponding baseline injected at the beginning (initial, upper panel) and at the end (24 h, bottom panel) of a sample sequence. The IgG electropherogram (blank trace) should be the product of superimposition of the peak profiles and the baseline (red trace). A section of the baseline is highlighted by yellow dotted vertical lines in order to demonstrate the shift of the waviness comparing between the top panel electropherogram and the bottom panel electropherogram [1], used with permission.

Despite CE‐SDS's benefits, irregular baselines hinder accurate electropherogram integration compared to chromatographic methods. These irregularities pose a significant obstacle for analysts and scientists seeking to assess the purity and stability of biopharmaceuticals. The integration process is often further complicated by the presence of broad peaks, incomplete resolution, and migration time shifts, making batch reprocessing and the application of a uniform integration method to all samples highly improbable. Figures 1 and 2 demonstrate how the baseline CE‐SDS manifests in at least three potential integration approaches. Figure 2 uses the yellow shaded area to estimate the error in each approach. Figures 1 and 2 only address the integration challenges due to the baseline drift and noise. However, another factor confounding auto‐integration is migration time reproducibility. In CE‐SDS, it has been reported that peak migration times can shift with successive analyses [1]. Similarly, the baseline features also shift. However, the baseline features shift at different rates from the peaks. As a consequence, auto‐integration is further hindered by the need to update timed‐events in the integration method for each analysis (Figure 3) [1].

In addition to CE‐SDS, peak integration is an equally relevant issue for other CE modes, such as capillary isoelectric focusing (cIEF), capillary zone electrophoresis (CZE), and micellar electrokinetic chromatography (MEKC).

Peak integration involves calculating the area of peaks. The concentration of substances the method separates is usually linearly related to their peak areas. The challenge in determining the area does not stem from the integration algorithm itself but from defining the integration limits, that is, the start and end of the peak. If there is baseline separation, a peak begins when the signal rises above the baseline and ends when the signal returns to the baseline. Although this may seem straightforward, the effectiveness of this process depends on the characteristics of the baseline and the peak. Integration can become particularly challenging when these characteristics are not ideal (refer to Section 2.1).

The integration of chromatograms is a foundational concept, tracing its roots to early practices in chromatography. Surprisingly, despite the pivotal role played by integration in analytical procedures (APs), authoritative sources such as the United States Pharmacopeia (USP) [2], the European Pharmacopoeia (Ph.Eur.) [3], and the Japanese Pharmacopoeia (JP) [4] offer limited guidance on proper integration techniques. The World Health Organization (WHO) guidance, “Good Chromatography Practices,” while emphasizing the need for automatic integration, falls short of providing specific directives for integration (see also Section 2).

Small molecules in gas and liquid chromatography typically produce narrow, symmetrical peaks and flat, stable baselines that can be easily integrated using standard chromatography data systems (CDS) integration functions, facilitating batch processing. However, this scenario changes when dealing with complex samples, for instance, large molecules such as mAbs. These larger entities, characterized by poor mass transfer and diffusion properties relative to small molecules, result in broader peaks, making automatic integration more challenging than for small molecules. Unlike chromatography, CE peaks are not expected to be symmetrical and instead are asymmetrical due to electromigration dispersion (EMD). In addition, proteins exist as the distribution of proteoforms, which likely have slight electrophoretic mobility differences, and this also contributes to broader peaks. The difficulty of distinguishing broad, asymmetrical peaks from the baseline highlights the complexities of integration practices, particularly for electropherograms.

This article addresses the complex factors impacting the integration of electropherograms, particularly irregular baselines, broad peaks, tailing and fronting peaks, and incomplete resolution (Figure 1). In addition, negative peaks can occur, for example, working with conductivity detection [5, 6] and literature cited therein. Prior to addressing these challenges, we will review the current regulations and expectations related to peak integration. Considering the existing gaps in guidance, we will explore the evolution of integration practices, modern CDS integration functions, and common myths and misconceptions about integration.

This article will also emphasize the necessity of manual integration in certain CE applications and how to address data integrity in GMP facilities to meet the expectations of regulatory inspections. The increased scrutiny of integration practices necessitates a comprehensive framework to assist analysts and regulators in understanding the challenges and expectations of electropherogram integration.

In subsequent sections, we will explore integration algorithms, baseline characteristics, and peak detection mechanisms in detail, providing a comprehensive perspective to pave the way for standardized integration practices in CE.

## 2 Authoritative Sources and Scientific Literature on Auto‐ and Manual Integration

### 2.1 Integrating Electropherograms

When creating policies and procedures to guide the integration of electropherograms, it must be understood that an electropherogram is fundamentally different from a chromatogram (see Sections 3 and 3.1.3 in particular). Chromatography software programs are designed with the expectation of Gaussian‐shaped peaks and flat, smooth baselines. However, CE does not conform to these expectations, often resulting in non‐Gaussian peaks and irregular baselines.

Although electropherograms do not fit the parameters that chromatography integration algorithms are based on, chromatography software is commonly used to analyze CE data. This practice stems from the visual similarity between electropherograms and chromatograms (i.e., a plot of response vs. time) and the business advantage of leveraging the widespread use of validated CDS to include the analysis of CE data. Challenges escalate when regulatory guidance for chromatographic data is applied to CE without considering how electropherograms differ from chromatograms. Therefore, it is imperative for CE practitioners to thoroughly understand the recent guidance documents related to integrating chromatograms and how these standards are being applied to the integration of electropherograms.

The use of time as the *x*‐axis for electropherograms is a common practice inherited from chromatographic techniques. However, this convention may not be the most effective for accurate data representation. Studies suggest that using electrophoretic mobility as the *x*‐axis provides a more precise measure for evaluating complexation constants and mobilities. One study highlights the limitations of using time as the *x*‐axis, emphasizing that the effective electrophoretic mobility of analytes provides a more accurate measure for evaluating complexation constants and mobilities, mitigating errors associated with EMD and complexation‐induced distortions [7]. Transforming the raw electropherograms with time on the *x*‐axis into an electropherogram with effective mobility on the *x*‐axis is highly effective in eliminating fluctuations in migration time caused by EOF changes, although it significantly alters the appearance of the electropherograms. Another study underscores the superiority of mobility‐based electropherograms over time‐based ones, arguing that the mobility scale offers enhanced reproducibility and robustness, particularly in the presence of fluctuating experimental conditions, thus providing a more reliable framework for analyte identification and quantification. These insights collectively advocate for a paradigm shift from time to mobility as the preferred *x*‐axis in electropherograms, enhancing the accuracy and reliability of CE data [8].

### 2.2 Pharmacopoeias and Guidance Documents

The issuance of the US FDA's guidance document “Data Integrity and Compliance with Drug CGMP: Questions and Answers” has led to a heightened focus on data integrity in regulatory inspections, particularly regarding the integration of chromatograms and electropherograms [9, 10]. In the past, the integration of chromatographic data has relied on an analyst's theoretical understanding of chromatography, their experience, and common sense. This knowledge, however, was rarely codified into written procedures, policies, and training materials to guide analysts and unify integration practices. Given the varying experience and integration skills of analysts, the development of formal integration procedures is a necessary improvement, and it is justified that integration practices are emphasized during inspections. GMP‐compliant companies must establish specific policies and procedures for integration and provide training that justifies integration practices and verification. Developing such a policy and training materials requires a thorough review of authoritative sources.

The FDA's data integrity guidance [9] provides definitions and expectations for audit trails in the context of chromatographic integration. An audit trail is a secure, computer‐generated, time‐stamped electronic record that reconstructs events relating to creating, modifying, or deleting an electronic record. The audit trail includes details such as the username, instrument setup, date and time of the analysis, the integration parameters used, and any reprocessing details, including the justification for reprocessing attempts. Furthermore, the guidance explicitly states that it is not acceptable to only save the final results from reprocessed data files. Complete data, including all results from reprocessed data, must be retained for review. Lastly, the guidance emphasizes that electronic data becomes a cGMP record as soon as it is created and cannot be modified without a record of the modification. The justification of reprocessing (i.e., reintegration) and the review of reprocessing is where many companies have fallen short of expectations. Several examples have been described [10, 11]. Official company procedures must inform when raw data will be integrated, how to perform that integration, provide expectations for integration practices, and provide examples of proper integration. Although the FDA guidance sets expectations for audit trails, it does not address integration approaches or best practices.

In addition to the FDA's guidance, the WHO provides guidance on data integrity, known as “TRS 1033 ‐ Annex 4: WHO Guideline on data integrity” [12]. This guidance document introduces the concepts of dynamic and static data in relation to chromatographic data. Dynamic data, such as electronic records, allow an interactive relationship between the user and the data. Chromatography records maintained as electronic records allow the user or reviewer to reprocess the data and expand the baseline for a clearer view of the integration. On the other hand, static data, such as article records, are fixed and allow little or no interaction between the user and the recorded content. They can no longer be reprocessed.

The concept of static and dynamic data is referenced in both the FDA and WHO data integrity guidance. Both documents indicate a regulatory preference for dynamic data, which aligns with the industry's use of CDS. Furthermore, companies are mandated to retain dynamic data records. The FDA and WHO data integrity guidance documents do not provide specific direction regarding performing integration and what constitutes acceptable and unacceptable integration practices. However, they serve as a starting point in outlining expectations for chromatography data, emphasizing the need for audit trails. “Reprocessing” (i.e., reintegrating chromatograms) is a vital component of both guidelines. The main takeaway is that companies must have written procedures that govern acceptable integration, document every instance of reprocessing, justify each reprocessing, require all data to be submitted for data review, and obtain reviewer approval of the integration acceptability.

Although the pharmacopoeias offer limited advice on integration, they do make some mention. For instance, the Ph.Eur. and JP prescribe “Measuring the signals from the detector as the peak area using a data processing system” [3, 4]. The USP recommends integrating the peak area of any impurity that is not completely separated from the main peak by tangential skimming [2].

The USP also highlights the significance of determining an appropriate reporting threshold and conditions for integrating peak areas, particularly when the related substances test sets a limit for total impurities or requires a quantitative analysis of an impurity. The pharmacopoeias harmonized general chapter on CE further advises that “when prescribed, the percentage content of one or more components of the sample under examination is calculated by determining the corrected area(s) of the peak(s) as a percentage of the total corrected areas of all peaks, excluding those due to solvents or any added reagents (normalization procedure)” [13]. Additionally, it recommends the use of an automatic integration system. The draft revision of this chapter further states that, with the exception of cIEF, peak areas are typically divided by the corresponding migration time to give the corrected area in order to compensate for the shift in migration time from run to run, thus reducing the variation of the response, and to compensate for the different responses of sample constituents with different migration times [14]. Collectively, these statements advocate for using a computerized data system for integration, which aligns with current industry practices.

Pharmacopoeias play a pivotal role in the pharmaceutical industry by setting quality standards. In addition, the WHOs technical report series provides invaluable insights and best practices to aid pharmaceutical product development, production, and quality control. Unlike pharmacopoeias, these reports are generally not legally binding. Nonetheless, these guidelines may be incorporated into the regulations by some countries’ regulatory authorities, thereby becoming legally enforceable within those jurisdictions. The WHO technical report, “Good Chromatography Practices,” provides a succinct overview of chromatographic data integration, offering general guidelines without going into great detail [12]. It reiterates the new standard of data integrity and emphasizes the importance of using validated methods, established conditions, and sound scientific practices to achieve symmetrical peaks. It also points out that it is essential to have fully separated peaks for accurate integration, and in cases where the peaks remain unresolved, the report recommends using a sound integration method. The guidance advocates the use of computerized systems. In a strong statement, the report points out that manual integration is only allowed in exceptional cases, provided that adequate records and authorizations are available. These guidelines seem to assume an ideal chromatography characterized by “symmetrical peaks” and “fully separated peaks.” However, they do not take into account the challenges inherent in separating biological macromolecules, such as proteins, and performing CE separations. Furthermore, this report lacks practical guidance on integrating chromatograms, let alone electropherograms.

### 2.3 Scientific Literature

The limited references to integration practices in the pharmacopoeias and the WHO report should not be perceived as a void of information on chromatographic integration. There is valuable information in the scientific literature. Although this literature does not carry the same authoritative weight as regulatory guidance documents, companies should strengthen their integration documents with insights from this literature to provide a solid scientific foundation (i.e., best practices).

One of the most comprehensive and influential books on chromatography is “Introduction to Modern Liquid Chromatography” by Snyder et al. [15]. It covers chromatographic theory, practice, and applications and, in doing so, offers guidance on integrating non‐ideal chromatograms and avoiding common integration errors. Those sections contain a limited number of practical illustrations that help to clarify proper integration. Snyder et al. refer to another book by Dyson, “Chromatographic Integration Methods,” [16], and quote his warning: “As long as integrators use perpendiculars and tangents and draw straight baselines beneath peaks, they are of use only in controlled circumstances when chromatography is good. Even then, the use of integrators requires vigilance from the operator and skill in assessing and assigning parameters.” This cautionary quote emphasizes that the essence of integration relies upon the skill and judgment of the operator. This stands in contrast to the misconception that integration software can operate independently of analyst input and decision‐making and the belief that auto‐integration is most accurate and inherently objective.

Dyson's “Chromatographic Integration Methods” provides a comprehensive and detailed study of chromatographic peak integration theory and practice [16]. The book is rich in illustrations demonstrating correct and incorrect integration, making it a valuable reference for integration procedures. Chapter 5, “Digital Measurement of Peak Areas,” explains the mathematical and physical intricacies of data acquisition and integration. This knowledge helps the analyst better understand the integration functions so that they can make informed adjustments to the integration parameters and functions, which in turn improves the accuracy and precision of the analytical results. This book is an essential reference and provides a solid foundation for organizations aiming to create SOPs, guidelines, and training materials for integration.

The book “Principles and Practice of Modern Chromatographic Methods” by Robards et al. features a chapter titled “Analysis,” which is supplemented with numerous informative figures. These include flow diagrams that illustrate the process from sample collection to final result and the data handling steps in chromatographic analysis. Such flow diagrams are a helpful addition to a general integration SOP and training materials [17, 18].

The Parenteral Drug Association (PDA) is a globally recognized provider of scientific, technological, and regulatory information to the pharmaceutical industry. As a practical voice, the PDA informs its members on critical issues impacting the industry and works with regulatory agencies such as the FDA and EMA to improve pharmaceutical science and technology in the interest of public health.

Perhaps the most detailed source on data integrity management for pharmaceutical laboratories is the PDA Technical Report No. 80, “Data Integrity Management for Pharmaceutical Laboratories” [19]. The PDA report, consistent with the regulatory guidance documents referenced earlier, points out the Quality Unit's obligation to create custom SOPs for data processing, each specific to a particular instrument or application. It underscores the need for a comprehensive audit trail to keep track of changes in processing parameters, methods, and processed data. Fortunately, the PDA report goes much deeper than the previously mentioned regulatory guidance documents by providing specific examples of proper and improper integration in the context of chromatography performed in GMP laboratories.

In Section 6.3.7, Data Processing and Peak Integration, the report provides instructions for integrating chromatograms using integration events such as peak width, threshold, height, and area and then visually inspecting the peak integration correctness. The report also recognizes that “inherent or analytical variations and the combination of various factors can lead to non‐Gaussian peaks and drifting baselines where the automatic integration either under‐ or over‐integrates the peak areas.”

By acknowledging the realities of non‐ideal chromatography and the crucial role of visual inspection, the authors adopt a more practical and realistic stance than the regulatory guidelines that assume ideal chromatography discussed above, but because they come from PDA, they have more authority compared to a book like Dyson's. This report is, therefore, an excellent resource for creating policies, procedures, and training material.

The PDA report advocates for auto‐integration as the preferred method of integration, defining it as the occurrence of consistent integration events across all chromatograms within a sample set or sequence. However, it acknowledges that imperfect chromatography may necessitate modifications and that variations could potentially cause auto‐integration to misrepresent peak areas. It also concedes that manual integration is sometimes unavoidable despite its time‐consuming nature and the need for skilled analysts.

In QC labs, the PDA report allows for manual integration to be performed under particular circumstances, such as complex chromatography due to sample matrix interferences, poor resolution, co‐elution of the peaks of interest, baseline problems, or software with limited capabilities. Furthermore, the report also points out that, occasionally, it is necessary to inhibit integration at specific points in the chromatographic run. The PDA acknowledges the common industry practice of using software to exclude peaks from solvents, mobile phases, placebos, and counter ions in impurity analysis, showcasing its practical approach to integration. Some official PDA reports include this peak inhibition as an integration parameter. The PDA emphasizes the crucial role of visual inspection in reviewing chromatograms to ensure accurate integration. Although CE methods are not mentioned in the report, as with non‐ideal chromatography, electropherograms present unique integration challenges due to baseline issues and irregularly shaped peaks, both of which are not readily accommodated by conventional CDS integration algorithms. The critical role of visual inspection is underscored, indicating that the discerning human eye, rather than CDS algorithms, ultimately determines what constitutes proper integration.

Robert D. McDowell has written numerous articles on chromatogram integration and, in several instances, challenges regulatory expectations based on the assumption of ideal chromatography. He shows that using a single auto‐integration method for all samples from a larger sequence is often impractical. McDowell's real‐world examples where auto‐integration is impractical are valuable for bridging knowledge gaps that may exist between bench scientists and quality assurance/management. Additionally, the illustrations in McDowell's articles are essential resources for companies developing SOPs, guidelines, and training materials for integration [11, 20, 21, 22, 23, 24, 25, 26, 27, 28, 29, 30].

### 2.4 A Company's or Laboratory's Internal Procedure

GMPs mandate the review and justification of integration settings, whether automated or manually integrated. The regulatory guidance documents mentioned above require that companies establish formal integration procedures, policies, and training programs. These procedures must be both in place and strictly adhered to, necessitating that they are practical and adaptable to effectively manage the complexities of integrating chromatograms and electropherograms. Although regulators clearly favor automated integration of entire chromatographic runs without analyst intervention, companies must rigorously assess the feasibility of this for each AP and ensure that provisions for manual integration are available in all overarching policy and procedural documents.

It is necessary to have a general SOP that provides detailed integration guidance and examples of correct integration. That SOP should be supported by an integration policy where the company commits to data integrity principles as they apply to integration and sets expectations for data analysis and review. Each AP, whether chromatography or CE, should be illustrated with examples of correct integration specific to that method. These illustrations provide a visual guide to ensure correct integration and, in turn, provide justification for final integration. Method validation chromatograms and electropherograms, particularly those from accuracy, range, intermediate precision, and robustness studies, offer crucial insights into complex integration scenarios, guiding analysts and justifying reviewers’ decisions. Additionally, examples from stability or forced degradation studies are valuable, as they present further challenging integration scenarios. Stability data, collected over extended periods, can capture the entire range of expected variability. This strategy, combined with an audit trail documenting the review process, will robustly support integration practices, whether automated or manual.

## 3 How to Properly Describe CE Integration

Manual peak integration of electropherograms and chromatograms has occasionally been classified as “Data Manipulation” by regulators and has led to Inspection citations and warning Letters [26, 31]. To avoid the appearance of unwarranted or malicious data manipulation, integration parameters must be documented, justified, and reviewed [9, 12, 32].

In theory, an ideal method would be robust enough to use consistent integration parameters across multiple runs, and the same integration parameters would be applied to all samples in a sequence [19]. However, integrating small, poorly resolved, negative, electromigration‐dispersed, or tailing peaks and shoulders are frequently too challenging for conventional CDS, necessitating significant time and expertise from the analyst to overcome these limitations. Due to complex sample matrices and challenging separations, most CE (and many chromatography) methods do not meet this ideal standard. Still, it is paramount that integration must be correct, regardless of the separation quality. Though preferred, if automatic sequential integration is incorrect for a subset of samples, resulting in under‐ or over‐reporting peak areas, the integration method must be adjusted for those samples. McDowell refers to this practice of using different integration parameters for different samples as “manual intervention” integration.

Repeatability experiments are crucial for assessing the precision of integration in separation science. Sample preparation is a significant source of error, second only to integration parameters. By repeatedly using the same solution (technical replicate), this source of error can be eliminated, allowing for a more accurate measurement of integration precision. Injecting the same stable solution multiple times and calculating the standard deviation provides a reliable estimate of integration error. Additionally, acquiring multiple measurements using various materials, reference materials, and calibration standards further enhances the accuracy of integration error estimation [33, 34, 35].

When accurate peak integration cannot be achieved with a single method for all samples in a sequence, the correct integration path should be clearly outlined in SOPs and/or APs. The path to final integration must be reviewed and justified in accordance with company procedures, and each iteration must be archived [11, 12]. Ultimately, it is more important to integrate peaks correctly than to do so automatically but incorrectly.

### 3.1 Data Collection and Integration Parameters

#### 3.1.1 Data Collection Parameters Prior to Integration

Before data can be integrated, it must be accurately collected, making the establishment of proper collection parameters essential. One crucial parameter is the sampling rate, measured in hertz, which indicates the number of data points collected per second. A Gaussian‐shaped peak requires approximately 20 data points for the integration algorithms to fit the peak accurately. Asymmetric peaks may necessitate more than 20 data points, depending on their shapes; therefore, the sampling rate should be developed accordingly.

Another important parameter is the bunching factor, also known as the smoothing factor, which averages several consecutive data points to create a time slice equivalent to a slower sampling rate. This reduces short‐term noise. Additionally, the correct wavelength for the analysis must be selected, which includes choosing an appropriate bandwidth. The bandwidth impacts signal quality by affecting noise levels, and many methods also utilize a reference wavelength to reduce both short‐term and long‐term noise. Properly set bandwidth settings help to ensure a smooth and flat baseline, facilitating better peak integration.

Although setting these data collection parameters is crucial, it is beyond the scope of this discussion, which focuses on integration. We assume that data collection parameters were optimized during method development. Our goal here is to outline the process of integrating an electropherogram after data collection.

#### 3.1.2 Integration Parameters: General Considerations

Typically, general integration parameters are included in a template CDS Table 1. A copy of this method is downloaded to a sequence for each data analysis, allowing for interventions to ensure proper integration based on the method's performance in that analytical setup. The template CDS method can be viewed as a starting point rather than a rigid set of conditions that must be adhered to (Note the contrast to the data collection parameters that are included in the AP and must stay the same from analysis to analysis). After separation is performed, the analyst evaluates the integration achieved by the template CDS method. The analyst assesses how to adjust the CDS method integration parameters to achieve proper integration, guided by the examples in the AP and established scientific principles. This may involve making minor adjustments to the template method, creating multiple data analysis methods for different sample subsets, and reintegrating the sequence with these methods assigned accordingly—a process McDowell referred to as “manual intervention” [11, 20, 22, 25, 36]. Alternatively, the analyst may have to resort to manually integrating by drawing baselines to achieve the most accurate integration.

The integration process should not be influenced by product specification. Instead, the focus must be on ensuring the accurate and appropriate integration of the electropherogram. Both the integration and its review should prioritize accuracy without regard to the final result.

**FIGURE 4:**
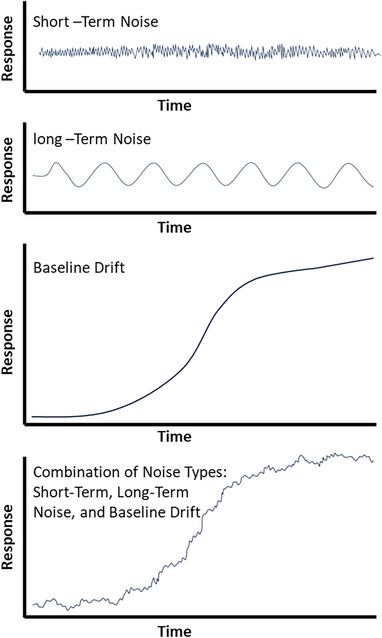
Various types of noise and baseline drifts that can hinder peak integration.

The critical aspect of integration is detecting where a peak starts and ends, defining the peak. Integration algorithms perform well in defining peaks when the peaks are large and baseline resolved and when baselines are smooth, flat, and exhibit minimal drift. When these conditions are not ideal (Figure 4), the analyst's expertise is required to adjust the parameters for proper integration. There are several common integration settings that can be fine‐tuned. Different CDS may use various names for these operations. Although the algorithms are designed to achieve a specific task, they may approach it in slightly different ways. Additionally, the desired effect can often be accomplished through different CDS functions. For example, instead of adjusting slope sensitivity settings, the baseline can be estimated by a spline function [37], and with the spline parameters optimized, the peak start and stop correctly determined peak, it makes sense to sort out all peaks below a threshold relevant to the method.

#### 3.1.3 Peak Beginning and End, Slope Sensitivity

**FIGURE 5:**
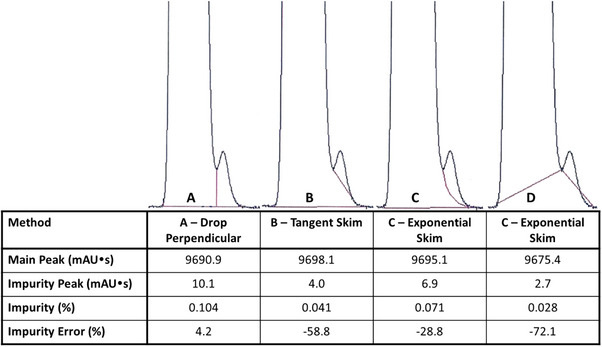
Integration of overlapping peaks of very different sizes with various peak integration functions (see Sections 2.1.8.3–2.1.8.5). A main peak and an impurity of 0.100 area% determined with four different integration modes. None of these integration modes return the correct value. Mode (A) (perpendicular drop) overestimates the impurity. Modes (B) (tangent skim), (C) (exponential skim), and (D) (valley to valley) underestimate the impurity. Often, modes (A) or (C) are closest to the true value, depending on the resolution and tailing of the main peak. The appropriate choice of integration mode depends on the intended purpose of the method. If this is a purity method for a drug substance of product, overestimation of the impurity generally is the safest approach for the patient.

A peak begins when the signal leaves the baseline and ends when it returns to it. Perhaps the most basic integration algorithm to identify a peak is slope sensitivity. Slope sensitivity and similar algorithms monitor the baseline's slope in small time intervals and compare the slope of the various time intervals to each other to detect slope changes. Although the peak ascends from the baseline, the algorithm tracks slope variations to identify critical points: an inflection point, the apex, and subsequently, the inflection point on the peak's descent back to the baseline. This algorithm works well when peaks are large and symmetrical, but it can be problematic for small peaks in trace analysis, peaks with shoulders, or when baselines are not ideal. When peaks in the electropherogram vary in size, this presents a challenge, as different sizes and shapes of peaks require different slope sensitivity settings (Figure 5 and Table 2). This means that different slope sensitivities may need to be set for different times in the electropherogram (see Section 3.1.8). For instance, if a peak (e.g., a product‐related impurity) is small in one sample and large in another, this creates a problem. A scenario like this is common when testing stability samples; a peak may be small at an early stability time point, and at a later time point, it might be much larger. As a result, if tested together in the same sequence, the slope sensitivity setting may properly integrate one sample and not the other.

Batch auto‐integration would be straightforward with sharp peaks that are well resolved, and the electropherograms have flat baselines. Unfortunately, many circumstances exist where these conditions are not met. Therefore, several other integration functions have been developed in various CDSs.

“Tailing peaks” and “fronting peaks” are non‐symmetrical peaks that pose challenges for accurate integration by CDSs and are characteristic of electrophoretic separations [38, 39, 40, 41]. CDSs offer a variety of functions that attempt to address this problematic phenomenon. These non‐symmetrical shapes can complicate integration because the traditional integration functions assume a symmetrical peak shape. The non‐symmetrical shape of tailing and fronting peaks can make it difficult for the CDS to establish a baseline from the start of the peak, especially if there is baseline drift or noise present in the electropherogram. This interference can further complicate the integration process and affect the accuracy of the results. In addition, CE peaks are commonly EMD [42, 43, 44, 45, 46, 47, 48, 49]. A typical EMD peak is characterized by its asymmetry, non‐Gaussian shape, and roughly triangular form, which is distinct from chromatographic tailing or fronting peaks. EMD peaks are challenging to integrate properly with the existing CDS software because the software was developed for chromatographic peaks where such shaped peaks would be highly improbable.

Software designed specifically for CE, such as CEVal, provides excellent support for CE peak integration. CEVal allows fitting distorted peaks caused by EMD using the Haarhoff‐van der Linde (HVL) function, which accurately describes the peak shape and migration time. This software provides comprehensive peak area information and related assessments, such as peak dispersion, which are crucial for CE analysis. Unlike chromatographic peak assessments, which often prove irrelevant for CE peaks, CEVal's tailored approach ensures precise and relevant evaluations for electropherograms [7].

#### 3.1.4 Peak Width

The Peak Width parameter, an indispensable integration setting along with slope sensitivity, assumes a critical role in accurately identifying and integrating peaks within CDS. Quantified in seconds, it estimates the anticipated width of a peak, thereby regulating the software's ability to discriminate peaks from baseline noise. This parameter is commonly an effective first line of approach in establishing the boundaries of a peak for integration purposes. Peak width, denoting the width of a peak at a specified height above the baseline, is typically assessed at a height between a peak's inflection points.

Evaluation of peak width often entails fitting a mathematical model, such as Gaussian or Lorentzian functions, to the peak shape and subsequently computing it based on the model's parameters. In certain CDS packages, such as Waters Empower, the Auto Peak Width feature adjusts it to 5% of the peak's maximum height in the second derivative, utilizing inflection point width and Gaussian peak width calculation. Adjustment of the Peak Width parameter enables the software to capture the entire peak accurately during integration, especially when peaks exhibit widened profiles due to phenomena like tailing or fronting. This ensures that the area or height of each peak reflects solely the response attributed to the analyte, minimizing the impact of background noise or drift. With electropherograms, the requirements for model fitting are often difficult to meet because they assume a Gaussian shape, thus further illustrating the challenging nature of integrating electropherograms.

#### 3.1.5 Smoothing

Data smoothing or filtering is typically performed during the data collection process before peak integration. Additionally, slope calculations (Section 3.1.3) include further smoothing or filtering after data collection.

CDS employ various “smoothing” functions to bolster the accuracy and precision of chromatographic data analysis. These functions augment the software's capacity to differentiate peaks from the baseline by mitigating noise. Smoothing is a processing step that reduces noise and fluctuations in the data, making it easier to detect and integrate peaks accurately. Smoothing algorithms help enhance the signal‐to‐noise ratio, making detecting and integrating peaks easier. Bunching is a common smoothing approach involving grouping or “bunching” together data points in an electropherogram.

A less commonly used but often included CDS smoothing function is Spline. Splines are mathematical functions used in CDSs to smooth chromatographic signals, reducing noise and baseline fluctuations. By fitting a smooth curve to the data, splines improve peak identification, correct baseline distortions, and enhance peak shape, ultimately leading to more accurate integration results.

In mathematics, a spline is a piecewise polynomial function used for interpolation and smoothing. It consists of connected polynomial segments, ensuring continuity at the transition points. Within chromatography, the spline algorithm (e.g., Empower's and Compass´s Spline fit [37]) is employed to interpolate data segments, thereby improving the adaptability of chromatograms generated from collected data points for integration.

##### 3.1.5.1 Baseline Correction

Baseline correction helps improve the accuracy of peak integration by ensuring that the baseline is adequately adjusted, leading to a more precise determination of peak heights and areas. This function is designed to minimize inaccuracies caused by baseline drift or noise, allowing for more reliable quantification of analytes in the sample. Baseline correction is often necessary because chromatograms and electropherograms can exhibit baseline drift, noise, or other irregularities that can affect peak integration accuracy (Figure 4, types of baseline noise). CDS software typically offers baseline correction functions that employ algorithms such as linear interpolation, polynomial fitting, or smoothing functions such as Savitzky–Golay or moving average filters. These algorithms analyze the data to identify and correct baseline deviations while preserving the integrity of the peaks.

#### 3.1.6 Thresholds

##### 3.1.6.1 Minimum Area/Peak Area Reject

Threshold functions set minimum and maximum thresholds for peak area and peak height and are valuable tools in the analysis of electropherograms. The minimum threshold typically refers to the smallest peak area or height that will be considered for integration. Peaks falling below this threshold are disregarded as noise. Conversely, the maximum threshold refers to the largest peak area or height that can be processed. Peaks exceeding this threshold may indicate detector saturation or other anomalies that could skew the data if not adequately addressed. Thresholds are not arbitrary but are carefully chosen on the basis of the sample's specific characteristics, the detector's sensitivity, and the test method's overall scope specific characteristics of the sample, the sensitivity of the detector, and the overall scope of the test method. These thresholds provide a means to filter out noise and manage anomalies.

#### 3.1.7 Shoulder Detect

A shoulder is a smaller peak that appears on the leading or trailing edge of another peak resulting from partial resolution of analytes where no valley between the analytes exists. Often, one analyte is in great excess, further impeding the resolution of the two analytes and obscuring the peak from the integration parameters mentioned above. These peaks can errantly be overlooked or merged with the larger peak, from which they are poorly resolved, during the integration process and are a significant challenge for auto‐integration programs to detect and integrate properly. However, they typically represent unique analytes that are important to quantify. The shoulder detect functions are designed to help identify such shoulder peaks and ensure they are properly accounted for during integration. These functions work by analyzing the peak shape (e.g., slope intervals) and identifying points where a change in slope indicates the presence of a shoulder peak. The sensitivity of the shoulder detect function can often be adjusted to match the characteristics of the sample and the separation method. This allows for a balance between detecting true shoulder peaks and avoiding false peaks from noise or minor fluctuations.

#### 3.1.8 Timed Events

Timed events are programmed actions or adjustments to integration parameters scheduled at specific migration times in the electropherogram. Because peaks can vary in size, shape, or resolution at different times, timed events allow for the application of tailored integration parameters to optimize accuracy. This approach provides a higher level of control over the integration process, contributing to improved accuracy and reliability of the results. The success of auto‐integration hinges on the precision of migration time. In fact, the inability of some CE methods to auto‐integrate, leading to a reliance on manual integration, can often be traced back to inadequate migration time precision.

##### 3.1.8.1 Inhibit Integration

The inhibit integration function allows switching off timed events and resuming integration at various time points within the electropherogram. Multiple phenomena warrant the use of inhibit integration, including peaks that fall outside the method's scope (such as excipients and matrix components) and baseline features unrelated to the sample (e.g., system peaks). As system peaks are inherent to CE and are more common than in chromatography, inhibit integration emerges as a crucial feature for the effective integration of electropherograms.

##### 3.1.8.2 Negative Peaks

Negative peaks that fall below the baseline are more prevalent in CE than in chromatography and pose a unique challenge in CE. Integration algorithms must be adept at managing these peaks to ensure accurate quantification.

The emergence of negative system peaks in electropherograms can be traced back to specific factors. These factors include discontinuities in the concentration of co‐ions and variations in the pH of the background electrolyte. Discontinuities throughout the electrophoretic system, spanning from the inlet vial to the sample plug, the separation capillary, and finally to the outlet vial, substantially impact peak behavior. These disparities, whether due to variations in concentration or pH, contribute to the appearance of system peaks. On the basis of the nature of the discontinuity, some of these peaks manifest as negative peaks or baseline “dips” [39, 40, 41]. If the CDS functions for negative peaks do not facilitate the removal of negative peaks from the integration, the “inhibit integration” function can be used.

##### 3.1.8.3 Split Peak

The “split peak” function in CDS software is designed to address situations where multiple peaks migrate together with only partial resolution, making it challenging to integrate the individual peaks accurately. The split peak function allows non‐baseline resolved peaks to be integrated as individual peaks. This division typically involves specifying the migration time at which the peak should be split and adding it to the integration table as a timed event. By splitting the peak, each component can be quantified separately despite not being entirely resolved. As with any timed event, migration time precision is necessary for the function to be used effectively.

##### 3.1.8.4 Drop Perpendicular

Similar to the split peak, the “drop perpendicular” function is particularly beneficial when the above integration parameters, such as “shoulder detect,” cannot integrate shoulders. This function uses a timed event to establish an integration border (i.e., peak start) at a specified time. “Drop perpendicular” is an integration strategy that typically overestimates the area of the shoulder. However, some laboratories prefer this approach as it is a conservative solution that errs on overestimating impurities rather than overestimating purity and, therefore, is less likely to be challenged.

##### 3.1.8.5 Tangent Skim

An alternative to drop perpendicular is the more sophisticated tangent skim function. The tangent skim function addresses this issue of unintegrated shoulders by applying a tangent line at the inflection point on the shoulder peak. The area demarcated by the tangent line (the “skimmed” area) is then subtracted from the total area under the peak. Depending on the resolution, this might result in peak areas more accurately representing the main and shoulder peaks relative to the drop perpendicular function (Figures 2 and 5).

#### 3.1.9 Selected Advanced Algorithms Unique to Commercial Software Package

##### 3.1.9.1 Agilent “Peak Recognition Filters”

Agilent's ChemStation CDS incorporates three peak recognition filters that operate by detecting changes in the slope and curvature within a continuous set of data points. These filters use the first derivative (to measure slope) and the second derivative (to measure curvature) of the examined data points. Effectively recognizing peaks based on these mathematical criteria by effectively recognizing peaks based on these mathematical criteria, it enhances peak detection accuracy.

##### 3.1.9.2 Chromeleon “Cobra”

The Cobra peak detection algorithm within the Thermo Scientific Chromeleon CDS aims to simplify peak integration and simplify data analysis processing to achieve more consistent and reliable peak detection across multiple analyses. It is meant to better address challenges such as distinguishing peaks from noise, identifying underlying baselines, and handling shouldered peaks than traditional algorithms and can enhance the reliability of quantification.

##### 3.1.9.3 Empower “Apex Track”

The ApexTrack integration function, part of Waters Corporation's Empower CDS, significantly improves traditional integration algorithms. Traditional algorithms rely on peak width and threshold to detect peaks from the baseline. ApexTrack employs a cubic spline algorithm and effectively detects and integrates shouldered peaks, providing more reliable detection of low‐level peaks on noisy or sloping baselines. This leads to less manual manipulation and more accurate results.

##### 3.1.9.4 CE‐Specific Software

Bio‐Techne´s Compass software and Sciex´s 32 Karat software offer similar features as the ones mentioned above. Compass also employs spline algorithms to adjust to more difficult baselines. Although initially a chromatographic software, 32Karat implemented the choice of using the Ceasar integration algorithm. This CE‐specific algorithm better integrates EMD peaks. However, the algorithm should not be used for broader peaks, such as for proteins in CE‐SDS. CEVal software (https://github.com/echmet/CEval), although open source and thus limited in its use within GMP environments, was specifically developed for integrating CE peaks. It addresses the unique challenges posed by non‐Gaussian peak shapes characteristic of CE, such as those distorted by EMD. Future revisions of 21CFR part 11 compliant software should incorporate similar approaches to effectively handle these non‐Gaussian peaks, ensuring accurate and reliable integration of electropherograms [7].

Table 1 categorizes and contrasts the integration functions of leading CDS and CE‐specific software. Perfect alignment of algorithms across different software is unattainable due to their unique programming. However, the objectives of these functions are often analogous, which plays a role in these groupings.

**TABLE 1:** Comparative list of integration functions provided by common chromatography data systems (CDS) software used for capillary electrophoresis.

Timed integration events	ChemStation	Chromeleon	Empower	32 Karat
Core integration functions	Slope Sensitivity	Sensitivity	Liftoff	Threshold
			Touchdown	
	Peak Width	Peak Slice	Peak Width	Width
	Auto Peak Width			
	Area Reject	Minimum Area	Minimum Area	Minimum Area
			Maximum Area	
	Height Reject	Minimum Height	Minimum Height	
			Maximum Height	
Integration functions for unresolved peaks	Shoulders Peak	Shoulder Threshold	Detect Shoulders	Shoulder Sensitivity
	Detect Shoulders			
	Shoulder Mode			
	Split Peak		Force Drop Line	Split Peak
	Tail Tangent Skim		Exponential Skim	Back Tangent Skim Front Tangent Skim
	Tangent Skim Mode		Tangential Skim	
	Baseline at Valleys	Valley to Valley	Valley to Valley	Valley to Valley
Integration functions for baseline corrections	Baseline Now	Lock Baseline		Move Baseline Start
	Baseline Hold	Lock Baseline		Move Baseline Stop
	Baseline Backwards		Forward Horizontal	Horizontal Baseline
	Baseline Next Valley		Reverse Horizontal	Backward Horizontal Baseline
			Force Baseline	Manual Baseline
			Force Peak	Manual Peak
Miscellaneous Integration functions	Integration	Inhibit Integration	Inhibit Integration	Integration Off
	Area Sum	Peak Group Start		Define Group
	Peak Group End			Define Group
	Solvent Peak			
	Negative Peak	Detect Negative Peaks	Allow Negative Peaks	Negative Peak
Advanced Peak Detection Algorithms	Peak Recognition Filters	Cobra	Apex Track	Caesar Integration

**TABLE 2:** Comparing peak data related to Figure 5.

Method	A—Drop Perpendicular	B—Tangent Skim	C—Exponential Skim	C—Exponential Skim
**Main peak (mAU s)**	9690.9	9698.1	9695.1	9675.4
**Impurity peak (mAU s)**	10.1	4.0	6.9	2.7
**Impurity (%)**	0.104	0.041	0.071	0.028
**Impurity error (%)**	4.2	−58.8	−28.8	−72.1

### 3.2 Manual Integration

**FIGURE 6:**
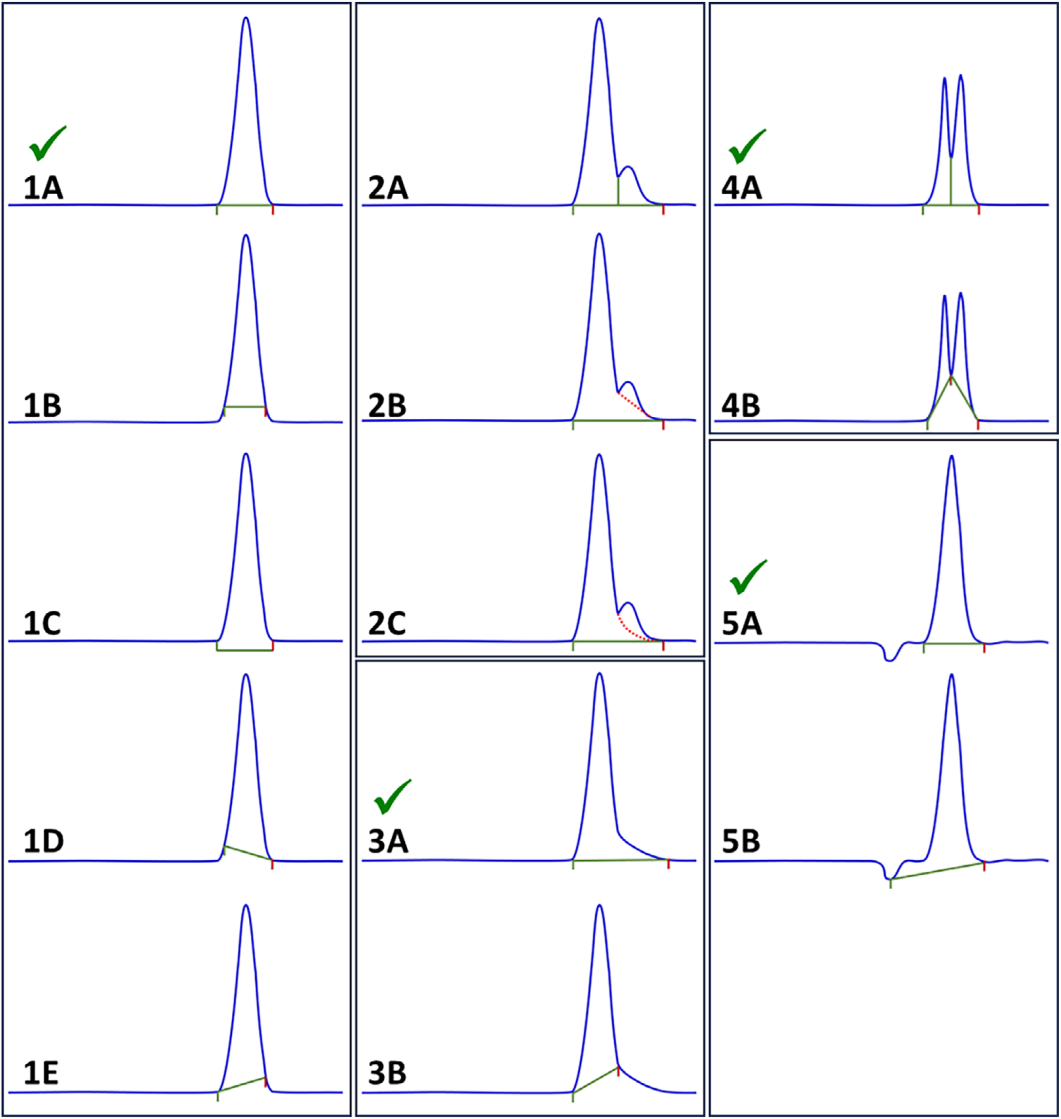
Examples of proper and improper integration. Panels 1A through 1E show the placement of integration start and stop points. 1A‐represents proper integration. 1B‐improper integration due to, for example, threshold, slope sensitivity, and/or peak with settings. 1C‐improper integration due to, for example, threshold, slope sensitivity, and/or peak with settings. 1D‐improper integration on the front side of the peak due to, for example, threshold, slope sensitivity, and/or peak with settings. 1E‐improper integration on the back side of the peak due to, for example, threshold, slope sensitivity, and/or peak with settings. Panel 2A through 2C show three ways a shoulder peak might be integrated. 2A‐a shoulder integrated using a drop perpendicular. This approach often overestimates the shoulders area. It is a common approach used when the shoulder is an impurity, and companies feel overestimating the impurity is more prudent then potentially underestimating it. 2B‐a shoulder integrated using tangent skim. Using this approach to integration should be supported with experimental data demonstrating that it is the most accurate approach. 2C‐a shoulder integrated using exponential skim. Like with tangent skim, this approach to integration should be supported with experimental data so demonstrating that it is the most accurate approach. Panels 3A and 3B show the integration when there is tailing of the peak: 3A‐a tailing peak where the tail is known to be the same substance as the rest of the peak and is integrated as one peak. 3B‐improper integration of a tailing peak where the tail is not included as a part of the peak. Panels 4A and 4B show integration of unresolved peaks. 4A‐a drop perpendicular integration of two closely migrating peaks with incomplete resolution. This is the most common approach used to integrate two close closely migrating peaks. Experimental data should be used to support this integration. 4B‐a baseline to valley integration approach. This is less common than the drop perpendicular integration and thus should be supported with experimental data. Panels 5A and 5B show integration when there is a closely migrating negative peak. 5A‐proper integration of a peak closely migrating after a negative system peak. 5B‐improper integration of a peak migrating after a negative system peak. Negative peaks add to the baseline complexity, often complicating the integration of peaks migrating near them.

Using the same parameters for autointegration ensures desirable consistency. However, incorrect integration is not in the patient's best interest. If fully automatic integration results in improperly integrated peaks for a given data set, manual integration becomes necessary. To ensure data integrity and traceability, it is essential to establish clear rules and procedures for manual integration. Although strict regulations govern the (bio)pharmaceutical industry, the positive aspect is that these guidelines require scientifically sound procedures (e.g., ICHQ7 part 11.12; CFR 21 part 11.160(b)). These regulations also require that integration remains consistent over time. To ensure this consistency, correct and incorrect integration examples should be documented in the AP (Figure 6, Examples of Integration).

None of the references mentioned above suggest banning manual integration. However, they emphasize the importance of providing scientifically sound data and justifying integration to ensure that data has not been manipulated to influence the outcome. Consistently implementing sound justification requires a robust integration policy and procedures, along with specific instructions in the AP. Analytical systems are subject to variability, leading to differing data characteristics from day to day or even within a single run. For instance, baseline noise and drift may vary across different instruments or on the same instrument as components, like UV lamps and capillary cartridges, age. Variations can also arise from sample types due to matrix differences and impurity levels. These examples highlight that it can be challenging, or even impossible, to auto‐integrate an entire batch of samples using a single integration method, especially in some CE methods.

As discussed earlier, the challenge for companies lies in developing effective policies and procedures that ensure consistent and justifiable integration results. This is especially critical when manual integration is involved, as it requires even greater rigor in documentation and training. A comprehensive SOP, applicable organization‐wide, should establish a general approach to integration while clearly outlining scenarios where manual integration is permitted. Each AP should include specific examples to guide integration for that procedure. Additionally, APs should specify that when automatic integration does not produce results consistent with the provided examples, manual integration becomes necessary.

Given the heightened importance of accuracy in manual integration, thorough training on scientifically sound peak integration principles and practices is essential for all analysts, reviewers, and supervisors involved in data processing and review. Scenario‐based training should include examples of common pitfalls, such as overestimating an impurity peak through drop valley integration or underestimating it using tangent skim integration. This training must ensure that individuals understand not only the technical aspects of integration but also the critical importance of precise documentation to maintain data integrity. The training program and formal documentation process must be clear and well understood to ensure transparency and accountability.

The term “manual integration” has been interpreted differently by individuals, companies, and agencies. In one interpretation, manual integration is defined as the manual repositioning of the baseline [21]. The PDA further defines it as the process by which a person manually adjusts the peak height or area by modifying the chromatogram baseline using chromatographic software [15]. However, both definitions overlook the requirement that integration must be accurate and scientifically sound. McDowall, in collaboration with Longden, refined the term, describing manual integration as “manual repositioning of the peak baseline with scientific justification for its positioning” [16]. Additionally, McDowall introduced the term “manual intervention” to differentiate it from manual integration. Manual intervention involves using specific integration settings for a subset of samples within an analytical run sequence to account for variations in the chromatogram or electropherogram. In CE, this often includes adjusting peak windows to compensate for migration time shifts. Processing parameters, or timed events, are then adjusted to enable proper auto‐integration for these sample subsets, applying different integration parameters as needed.

Finally, it is important to recognize that good integration practices cannot compensate for poor separations even with CE methods. If peak integration continues to be problematic, further method development should be carefully considered [15, 16, 50].

### 3.3 Reporting Integration

The documentation of integration parameters is as crucial as other experimental parameters, given their significant influence on the results of quantitative methods (refer to Sections 3.2 and 3.3 for comparison). These parameters should be meticulously specified in the experimental sections of publications, as is expected of APs. The parameters outlined in Section 2.1 and Table 1 can serve as a reference for determining relevant parameters. Any additional parameters utilized should also be clearly specified (e.g., scale used during integration).

Various algorithms can estimate several parameters, such as slope and smoothing. These include polynomial fitting, Savitzky–Golay, moving average filters, and various spline algorithms (refer to Sections 3.1.5 and particularly 3.1.5.1 for details). It is most effective to specify the algorithm used explicitly and indicate the software version to clarify the algorithm employed.

In instances where manual integrations are required, they should be detailed comprehensively. Whether the integration is manual or automated, including examples of integrated signals in the AP is highly recommended. Tracking the auto‐integrations and manual integration frequencies would be advantageous to identify trends. In publications, providing raw data as supporting or supplementary information is also strongly advised.

## 4 Conclusions

The regulatory guidance documents referenced here primarily address chromatography and typically do not specifically mention CE. CE is typically chosen as a separation mode when it offers superior separation relative to chromatography, which, in turn, offers greater accuracy. In these cases where CE provides superior separation, the samples are typically more complex, and analytical tasks are more challenging. Electropherogram baselines often present unique challenges that may push the boundaries of chromatography integration algorithms. Consequently, the integration process for CE is notably more intricate compared to chromatography, and regulatory guidance documents (e.g., pharmacopoeias) have not incorporated those realities.

Due to its speed and objectivity, automatic integration is favored by regulatory bodies and serves as a more efficient workflow for laboratories. It should be the preferred method, and consequently, method development should be dedicated to facilitating routine auto‐integration. Similarly, AP training should incorporate a concentrated focus on integration. Regulatory guidance documents clearly express a preference for automatic integration over manual methods. However, manual integration is permitted by these guidances. Nevertheless, regardless of whether automatic or manual integration is utilized, it is necessary that companies establish a policy articulating their approach and principles regarding integration as it applies to data integrity. Furthermore, it is essential for companies to establish SOPs delineating the criteria for proper integration and identifying instances of improper integration (see Section 2.4). Individual APs and experimental sections in publications should include all relevant parameters for detection (see Section 3.1.1) and the relevant integration parameters (see Table 1). The AP should also include scenarios necessitating integration in diverse, challenging separations. Manual integration should be kept to a minimum but should remain permissible if required. Therefore, it should be described as well as possible. Additionally, each AP should incorporate detailed examples illustrating the integration of electropherograms, serving as references for analysts and data reviewers. Ultimately, given that integration proficiency is an essential competency for analysts, it is imperative to implement a comprehensive training program for analysts, data reviewers, and approvers.

Differences in integration software present a challenge in developing effective guidance. Establishing benchmark datasets for comparing various software packages would help address this issue. Software vendors are encouraged to update and enhance their products, incorporating advances in artificial intelligence to reduce the reliance on manual integration.

We call on industry leaders to unite to form a consortium dedicated to creating a comprehensive guideline for integrating electropherograms in CE.

## Conflicts of Interest

The authors declare no conflicts of interest.

## Data Availability

The data that support the findings of this study are available from the corresponding author upon reasonable request.
